# Self-Reported Physical Activity Levels in Patients With Arthritis: A Retrospective Study

**DOI:** 10.7759/cureus.74239

**Published:** 2024-11-22

**Authors:** Sriram Ravichandran, Parth P Patel, Renee Manake, Rahul Sharma

**Affiliations:** 1 General Practice, PSG Institute of Medical Sciences and Research, Coimbatore, IND; 2 General Practice, Kharkiv National Medical University, Kharkiv, IND; 3 Internal Medicine, C U Shah Medical College and Hospital, Surendranagar, IND; 4 Internal Medicine, School of Medicine, Makerere University College of Health Sciences, Kampala, UGA; 5 Orthopedics, Jawaharlal Nehru Medical College and Hospital, Bhagalpur, IND

**Keywords:** arthritis and orthopaedic rheumatology, arthritis and physical activity, brfss database, chronic gout, exercise and rheumatology, lupus and exercise level, physical activity level, public health intervention, rheumatoid arthritis, socio-economic factors

## Abstract

Introduction

Arthritis affects a significant number of adults in the United States, leading to pain and limited mobility. This study explores the impact of physical activity on patients with arthritis, including rheumatoid arthritis, gout, lupus, and fibromyalgia. Using data from the Behavioral Risk Factor Surveillance System (BRFSS), it examines how exercise may improve symptoms and quality of life for these patients.

Aim

The aim of this study is to evaluate the prevalence of self-reported physical activity among US patients with arthritis, rheumatoid arthritis, gout, lupus, or fibromyalgia while considering demographic, socioeconomic, and healthcare access variables.

Methodology

A retrospective study was conducted using 2021 data from the BRFSS, analyzing responses from 434,985 participants. The focus was on arthritis diagnosis and physical activity, with variables including demographics, socioeconomic status, and healthcare access. Statistical analysis was performed using cross-tabulation, chi-square tests, and Fisher’s exact test.

Results

In 2021, 434,985 individuals participated in the BRFSS study, with 32.69% reporting a diagnosis of arthritis, rheumatoid arthritis, gout, lupus, or fibromyalgia. Among these patients, 68% engaged in physical activity, compared to 79.3% of those without these conditions. Physical activity levels varied significantly across factors such as age, gender, race, education, employment, income, and recent medical checkups.

Conclusions

This study found that 68% of participants with arthritis-related conditions were physically active, with activity levels varying across demographic and socioeconomic factors. Younger individuals, males, White non-Hispanics, and those with higher education, income, and employment were more likely to be active. Additionally, regular medical checkups were associated with higher physical activity levels, underscoring the need for targeted interventions to improve activity in underserved groups.

## Introduction

Arthritis is the inflammation of one or more joints, leading to pain, swelling, warmth, erythema, and limited range of motion. The estimated prevalence of arthritis in US adults aged ≥18 years was 21.2% (18.7% age-standardized) between 2019 and 2021, affecting approximately 53.2 million people [1]. As a chronic condition, arthritis can significantly impair joint mobility and reduce the ability to perform daily activities, resulting in negative physical, socioeconomic, and psychological consequences for affected individuals [2].

This study examines physical activity levels in patients with arthritis-related conditions, including osteoarthritis, rheumatoid arthritis, gout, lupus, and fibromyalgia. While the etiologies of these conditions differ - osteoarthritis being a degenerative process, rheumatoid arthritis and lupus involving autoimmune mechanisms with systemic and intra-articular manifestations, gout caused by uric acid crystal deposition, and fibromyalgia being a multifactorial syndrome [3,4] - all share a common pathophysiological feature: joint inflammation. Increased physical activity has been shown to reduce pain and improve joint range of motion, potentially mitigating disease progression through an anti-inflammatory response [5].

Pharmacological treatments, including disease-modifying antirheumatic drugs for conditions like rheumatoid arthritis and lupus, are effective in symptom management and disease progression. However, for osteoarthritis and fibromyalgia, physical activity serves as an effective, cost-efficient, and accessible approach to both treatment and prevention [6,7]. Incorporating regular exercise can improve the quality of life, alleviate symptoms, and slow disease progression in these patients.

The Behavioral Risk Factor Surveillance System (BRFSS) is a telephonic survey tool used in the US to gather data on health-related behaviors, chronic conditions, and preventive services. Using BRFSS data, this study aims to assess the impact of physical activity on individuals diagnosed with arthritis, rheumatoid arthritis, gout, lupus, or fibromyalgia.

## Materials and methods

This retrospective original research study was conducted using data from the BRFSS database [8]. The BRFSS is a state-based system of health-related telephone surveys in the US, collecting data on health-related risk behaviors, chronic conditions, and preventive service utilization among US residents. The data for this study were extracted from the BRFSS database on June 25, 2024. Since the BRFSS database is publicly available, contains anonymized data, and does not involve direct participation of human subjects, no ethics committee approval was required. The study utilized data from the BRFSS Web-Enabled Analysis Tool (BRFSS-WEAT) for the year 2021. The dataset included responses from 434,985 participants who completed the survey.

The primary disease variable analyzed was “Ever told you had some form of arthritis, rheumatoid arthritis, gout, lupus, or fibromyalgia?” (HAVARTH5), while the physical activity variable of interest was “During the past month, any physical activity or exercises such as running, calisthenics, golf, gardening, or walking for exercise?” (EXERANY2). Cross-tabulation was performed between the variables HAVARTH5 and EXERANY2 to assess the prevalence of physical activity among participants.

Several control variables were included in the study. Demographic variables consisted of age, categorized into four groups (_AGE_G): 18-24, 25-44, 45-64, and 65+. Gender (SEX1) was categorized as male or female. Race was classified into four groups (_RACEGR3): White non-Hispanic, Black non-Hispanic, Hispanic, and other. The demographic variables were cross-tabulated with the prevalence of self-reported arthritis, rheumatoid arthritis, gout, lupus, or fibromyalgia by physical activity level to determine how demographic characteristics influenced physical activity levels.

Socioeconomic variables included education level (EDUCA), which was divided into two groups: (a) basic education (never attended school or attended kindergarten, elementary grades 1-8, some high school, or high school graduate/GED) and (b) advanced education (some college or technical school, or college graduate). Employment status (EMPLOY1) was categorized as (a) employed (employed for wages or self-employed) and (b) not employed (out of work for one year or more, out of work for less than one year, or homemaker). Income (INCOME3) was divided into three groups: low income (<$50,000), middle income ($50,000-$150,000), and high income (>$150,000). The socioeconomic variables were cross-tabulated with the prevalence of self-reported arthritis, rheumatoid arthritis, gout, lupus, or fibromyalgia by physical activity level to analyze how socioeconomic factors affected physical activity.

The healthcare access variable was the time since the last routine checkup (CHECKUP1), categorized as (a) last checkup within the past year (one to 12 months) and (b) last checkup more than a year ago or never (including within the past two years, five years, or never). The variables HAVARTH5 and EXERANY2 were cross-tabulated with the control variable CHECKUP1 to assess the impact of healthcare access on physical activity levels.

Data were selectively retained according to the study's inclusion criteria, with irrelevant data excluded. Descriptive statistics were generated for each variable using cross-tabulations in the BRFSS Web-Enabled Analysis Tool. Data were stored in Microsoft Excel (Microsoft Corporation, Redmond, WA, USA), and statistical analyses were performed using R version 4.3.1 (R Foundation for Statistical Computing, Vienna, Austria). The chi-square test and Fisher’s exact test were employed for statistical testing.

## Results

In 2021, 434,985 individuals participated in the BRFSS study. Of these, 142,205 (32.69%) reported having some form of arthritis, rheumatoid arthritis, gout, lupus, or fibromyalgia (HAVARTH5) and were categorized as having one of these conditions.

Table 1 presents the prevalence of physical activity among the study participants. In the past month, 96,682 (68%) individuals with arthritis, rheumatoid arthritis, gout, lupus, or fibromyalgia engaged in physical activity, while 45,523 (32%) did not. Among those without these conditions, 232,156 (79.3%) were physically active, and 60,624 (20.7%) were not. The chi-square test indicated that physical activity was significantly associated with a lower prevalence of self-reported arthritis, rheumatoid arthritis, gout, lupus, or fibromyalgia.

**Table 1:** Prevalence of self-reported arthritis, rheumatoid arthritis, gout, lupus, or fibromyalgia by physical activity level Values are presented as n (%). ^*^ A p-value < 0.05 is considered statistically significant.

Parameters		During the past month, any physical activity or exercises such as running, calisthenics, golf, gardening, or walking for exercise? (EXERANY2)		p-value (chi-square test)
		Yes	No	
Ever told you had some form of arthritis, rheumatoid arthritis, gout, lupus, or fibromyalgia?(HAVARTH5)	Yes	96,682 (68%)	45,523 (32%)	<0.001^*^
	No	232,156 (79.3%)	60,624 (20.7%)	

Table 2 presents the prevalence of arthritis, rheumatoid arthritis, gout, lupus, or fibromyalgia by physical activity level, stratified by demographic characteristics of the study participants. The highest levels of physical activity among patients with these conditions were observed in the 18-24 years age group (81.9%, n = 665), males (71.9%, n = 39,963), and white non-Hispanic participants (69.2%, n = 78,532). Across demographic variables, physical activity was significantly associated with a lower prevalence of arthritis, rheumatoid arthritis, gout, lupus, or fibromyalgia in age groups 25-44 years, 45-64 years, and 65+, as well as among both males and females, and across racial groups, including white non-Hispanic, black non-Hispanic, Hispanic, and other races.

**Table 2:** Prevalence of self-reported arthritis, rheumatoid arthritis, gout, lupus, or fibromyalgia by physical activity level based on demographic characteristics of study participants Values are presented as n (%). ^*^ A p-value < 0.05 is considered statistically significant.

Variables	Arthritis, rheumatoid arthritis, gout, lupus, or fibromyalgia	N	Physically active	Physically not active	p-value (Fisher’s exact test)
Age groups					
18-24 years	Yes	812	665 (81.9%)	147 (18.1%)	0.177
	No	25,059	20,978 (83.7%)	4,081 (16.3%)	
25-44 years	Yes	12,184	9,172 (75.3%)	3,012 (24.7%)	<0.001^*^
	No	93,330	76,797 (82.3%)	16,533 (17.7%)	
45-64 years	Yes	50,261	34,358 (68.4%)	15,903 (31.6%)	<0.001^*^
	No	99,322	78,802 (79.3%)	20,520 (20.7%)	
65+ years	Yes	78,948	52,487 (66.5%)	26,461 (33.5%)	<0.001^*^
	No	75,069	55,579 (74%)	19,490 (26%)	
Gender					
Male	Yes	55,545	39,963 (71.9%)	15,582 (28.1%)	<0.001^*^
	No	146,413	118,200 (80.7%)	28,213 (19.3%)	
Female	Yes	86,660	56,719 (65.5%)	29,941 (34.5%)	<0.001^*^
	No	146,367	113,956 (77.9%)	32,411 (22.1%)	
Race					
White, non-Hispanic	Yes	113,547	78,532 (69.2%)	35,015 (30.8%)	<0.001^*^
	No	206,765	167,861 (81.2%)	38,904 (18.8%)	
Black, non-Hispanic	Yes	10,539	6,433 (61%)	4,106 (39%)	<0.001^*^
	No	21,995	16,261 (73.9%)	5,734 (26.1%)	
Hispanic	Yes	6,743	4,102 (60.8%)	2,641 (39.2%)	<0.001^*^
	No	31,334	22,064 (70.4%)	9,270 (29.6%)	
Other	Yes	8,028	5,363 (66.8%)	2,665 (33.2%)	<0.001^*^
	No	25,473	20,230 (79.4%)	5,243 (20.6%)	

Table 3 presents the prevalence of self-reported arthritis, rheumatoid arthritis, gout, lupus, or fibromyalgia by physical activity level, categorized by the socioeconomic characteristics of study participants. The highest levels of physical activity among patients with these conditions were observed in those with advanced education (73.7%, n = 69,055), high income (87.2%, n = 7,176), and those employed (76%, n = 35,431). Regarding socioeconomic factors, physical activity was significantly associated with a lower prevalence of arthritis, rheumatoid arthritis, gout, lupus, or fibromyalgia across both education levels (basic and advanced), employment status (employed vs. not employed), and income categories (low, middle, or high).

**Table 3:** Prevalence of self-reported arthritis, rheumatoid arthritis, gout, lupus, or fibromyalgia by physical activity level based on socioeconomic characteristics of the study participants Values are presented as n (%). ^*^ A p-value < 0.05 is considered statistically significant.

Variables	Arthritis, rheumatoid arthritis, gout, lupus, or fibromyalgia	N	Physically active	Physically not active	p-value (Fisher’s exact test)
Education level					
Basic education	Yes	47,928	27,253 (56.9%)	20,675 (43.1%)	<0.001^*^
	No	88,228	59,727 (67.7%)	28,501 (32.3%)	
Advanced education	Yes	93,706	69,055 (73.7%)	24,651 (26.3%)	<0.001^*^
	No	202,872	171,189 (84.4%)	31,683 (15.6%)	
Employment status					
Employed	Yes	46,625	35,431 (76%)	11,194 (24%)	<0.001^*^
	No	175,871	145,183 (82.6%)	30,688 (17.4%)	
Not employed	Yes	93,625	59,950 (64%)	33,675 (36%)	<0.001^*^
	No	110,949	82,450 (74.3%)	28,499 (25.7%)	
Annual income					
Low-income	Yes	58,073	34,408 (59.2%)	23,665 (40.8%)	<0.001^*^
	No	91,358	64,443 (70.5%)	26,915 (29.5%)	
Mid-income $50,000 to $150,000	Yes	45,513	35,104 (77.1%)	10,409 (22.9%)	<0.001^*^
	No	108,671	92,252 (84.9%)	16,419 (15.1%)	
High-income >$150,000	Yes	8,232	7,176 (87.2%)	1,056 (12.8%)	<0.001^*^
	No	30,369	27,915 (91.9%)	2,454 (8.1%)	

As shown in Table 4, among participants who had their last routine checkup within the past year, 67.5% (n = 83,154) of those with arthritis, rheumatoid arthritis, gout, lupus, or fibromyalgia were physically active, compared to 79.1% (n = 167,210) of participants without these conditions. This difference was statistically significant. Among participants who had never had a checkup or had one more than a year ago, 72% (n = 12,804) of those with arthritis, rheumatoid arthritis, gout, lupus, or fibromyalgia were physically active, compared to 80.4% (n = 61,904) of participants without these conditions. Regardless of the time since their last routine checkup, physical activity in the past month was significantly associated with a lower prevalence of arthritis, rheumatoid arthritis, gout, lupus, or fibromyalgia.

**Table 4:** Prevalence of self-reported arthritis, rheumatoid arthritis, gout, lupus, or fibromyalgia by physical activity level based on time since the last routine checkup Values are presented as n (%). ^*^ A p-value < 0.05 is considered statistically significant.

Time since the last routine checkup	Arthritis, rheumatoid arthritis, gout, lupus, or fibromyalgia	N	Physically active	Physically not active	p-value (Fisher’s exact test)
Within the past year	Yes	123,127	83154 (67.5%)	39,973 (32.5%)	<0.001^*^
	No	211,458	167,210 (79.1%)	44,248 (20.9%)	
More than a year ago or never	Yes	17,789	12,804 (72%)	4,985 (28%)	<0.001^*^
	No	77,016	61,904 (80.4%)	15,112 (19.6%)	

## Discussion

A retrospective study was conducted on June 20, 2024, to assess physical activity levels among US patients with arthritis in 2021, using the BRFSS database. Physical activity offers numerous benefits for arthritis patients, including reduced pain and improved function. Regular exercise strengthens muscles, enhances joint flexibility, and reduces stiffness. To achieve meaningful improvements in pain and function, the CDC recommends at least 150 minutes of moderate-intensity aerobic exercise per week, along with muscle-strengthening activities [9]. The Arthritis Foundation emphasizes that exercise helps manage arthritis symptoms by maintaining healthy joints, reducing inflammation, and improving overall fitness [10]. These findings underscore the importance of promoting physical activity as a key strategy in arthritis management.

Our study found that 68% (n = 96,682) of participants with self-reported arthritis, rheumatoid arthritis, gout, lupus, and fibromyalgia engaged in physical activity in the past month, compared to 79.3% (n = 232,156) of those without these conditions. Conversely, 32% (n = 45,523) of individuals with these conditions were inactive, while only 20.7% (n = 60,624) of those without these conditions did not participate in physical activity. A chi-square test indicated a significant association between physical activity and arthritis-related conditions. This aligns with the findings of Fontaine et al. (2004), who reported that individuals with rheumatoid arthritis are less likely to meet physical activity guidelines due to barriers like pain and fatigue [11]. Similarly, Murphy et al. (2017) found that adults with arthritis are 50% more likely to be inactive compared to those without arthritis [12]. These results highlight the ongoing challenge of promoting physical activity among arthritis patients to manage symptoms and improve their quality of life.

Physical activity was highest among patients with arthritis, rheumatoid arthritis, gout, lupus, or fibromyalgia in the 18-24 years age group (81.9%), males (71.9%), and White, non-Hispanic individuals (69.2%). Significant associations were found across all age groups, genders, and racial categories, with younger age, male gender, and White, non-Hispanic race linked to higher activity levels. In a similar study, Callahan et al. (2015) reported lower activity levels in older adults with arthritis due to barriers like pain and lack of motivation [13]. Additionally, Dunlop et al. (2017) found that women with arthritis have lower activity levels than men [14]. Our study underscores the need for targeted interventions to increase physical activity among older adults, women, and minority groups with arthritis-related conditions.

Regarding the influence of socioeconomic factors on physical activity levels, our study found the highest physical activity levels in participants with advanced education (73.7%), high income (87.2%), and employment (76%). There was a statistically significant association between physical activity and arthritis-related conditions across all education, employment, and income categories. These findings are consistent with Yelin et al. (2016), who reported that individuals with higher education and income are more likely to engage in physical activity due to better access to resources and opportunities [15]. Furthermore, Lorig et al. (2001) reported that employed individuals with arthritis are more likely to maintain physical activity due to structured daily routines and access to workplace wellness programs [16]. Our study highlights the importance of addressing socioeconomic disparities to promote physical activity among patients with arthritis-related conditions, emphasizing the need for targeted interventions for individuals with lower education, income, and employment status.

Among participants who had a routine checkup within the past year, 67.5% (n = 83,154) with arthritis-related conditions were physically active, compared to 79.1% (n = 167,210) without these conditions, a statistically significant difference. Among those who had never had a checkup or had one more than a year ago, 72% (n = 12,804) with arthritis-related conditions were active, compared to 80.4% (n = 61,904) without. Regular checkups were associated with higher physical activity levels, but individuals with arthritis-related conditions remained less active. This finding is consistent with Shih et al. (2015), who found that regular medical consultations positively influence physical activity through ongoing motivation and advice [17]. This underscores the importance of regular checkups in encouraging physical activity among those with arthritis-related conditions.

Limitations

This study has several limitations that should be considered when interpreting the results. First, the data were collected through a telephone survey, which may have excluded individuals without landlines or those who did not use them. The survey was also conducted in a limited number of languages, potentially excluding non-speakers. Additionally, the reliance on self-reported data, rather than clinical assessments, introduces the possibility of inaccuracies.

The dataset also lacks information on the type, severity, or duration of arthritis and related conditions, factors that could influence physical activity levels. Moreover, the survey focused solely on physical activity in the past month, which limits the understanding of its long-term effects. Finally, as a retrospective observational study, the analysis can identify associations but cannot establish causality.

## Conclusions

This study examined physical activity levels among US participants with arthritis-related conditions, revealing that 68% were active in the past month. Higher activity levels were observed among younger individuals, males, White non-Hispanics, those with higher education and income, and employed participants. Regular checkups were also associated with increased activity. These findings highlight the need for targeted interventions that consider demographic and socioeconomic factors. Despite limitations such as the retrospective design and reliance on self-reported data, future prospective studies are needed to better understand the impact of physical activity on these conditions, ultimately informing the development of guidelines to improve patient outcomes.
